# Rare congenital Dyserythropoietic anemia of childhood: A case report

**DOI:** 10.1002/ccr3.6975

**Published:** 2023-02-15

**Authors:** Hamzeh F. Al Hussien, Basil N. Al‐Ekeer, Hashem Abu Serhan, Issam Haddadin, Abdulqadir J. Nashwan

**Affiliations:** ^1^ Department of Pediatrics Islamic Hospital Amman Jordan; ^2^ Department of Ophthalmology Hamad Medical Corporation Doha Qatar; ^3^ Department of Nursing Hamad Medical Corporation Doha Qatar

**Keywords:** anemia, bone marrow, case report, CDA, congenital dyserythropoietic anemia, erythropoiesis, pediatrics

## Abstract

Congenital dyserythropoietic anemias (CDA) is a heterogeneous class of anemia of varying degrees of ineffective erythropoiesis and secondary hemochromatosis. We reported a case of CDA and showed our approach to reaching a diagnosis, highlighting the importance of the typical morphological appearance of bone marrow erythroblasts to reach the diagnosis.

## Graphical abstract

To avoid delay in diagnosing CDA and provide early care and better management, we urge clinicians to have a high degree of suspicion for congenital anemias when there is evidence of ineffective erythropoiesis in a child.

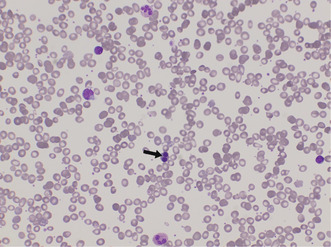

## 1 INTRODUCTION

Congenital dyserythropoietic anemia (CDA) with online mendelian inheritance in man (OMIM entry: 224100) is an uncommon hematological condition reported primarily in Central and Western Europe and North Africa.^1^, ^2^, ^3^ The hallmark of the disease is ineffective erythropoiesis as the main feature, and there are distinct morphological abnormalities of the bone marrow's erythroblasts. Therefore, it should be considered in any patient with chronic anemia. It is divided into three categories (CDA I, CDA II, and CDA III), with the most common type II and CDA III, whose nonfamilial type is the rarest. A majority of CDA cases are autosomal recessive in nature.^4^ In our case, we showed the way we used to reach our diagnosis and that the typical morphological appearance of bone marrow erythroblasts is considered the cornerstone of the diagnosis. Still, a blood smear might give us a hint.

## 2 CASE PRESENTATION

A 6‐year‐old female patient presented with chronic anemia for evaluation. She was doing well until the age of 3 years when the mother started to notice gradual and progressive pallor. It was associated with lethargy and intolerance to exercise with attacks of palpitation. Since then, the patient has been treated as a case of hemolytic anemia with regular blood transfusion every 3 months.

The patient is the product of vaginal delivery, term, and no neonatal intensive care unit (NICU) admission. The patient had two previous admissions for chest infections before the age of 3 years. The patient had developmental dysplasia of the hip (DDH). The patient is from Jordan with Hispanic ethnicity.

The parents are consanguineous (first‐degree). The patient has one younger healthy sister. No family history of chronic illnesses or splenectomy.

On examination, the patient had frontal bossing and mild maxillary hypertrophy with depression of the nasal bridge. The spleen was felt 6 cm below the costal margin. Table 1 showed the lab results of the patient.

**TABLE 1:** Basic laboratory results of our patient.

Labs	Values	Normal range	
HB(g/dl)	8.3	11.5–13.5	Low
PCV	25%	34–40%	Low
WBC (×10^3^)	7.5	5.0–15.5	Normal
Neutrophils	40.5%	40–75%	Normal
Lymphocytes	49.7%	20–45%	Normal
RBC (×10^6^)	2.35	3.3–4.9	Low
MCV	106	75–86	High
MCH	35	24.6–30.9	High
MCHC (g/dl)	33	31.0–37.0	Normal
RDW %	24.1%	11.8–14.9%	High
Reticulocytes	22.3%	0.5% ‐ 2%	High
Direct Coombs test	Negative	Negative	Normal
Ferritin (ng/ml)	487.5	7–140	High
LDH	385	60–170	High
Creatinine (mg/dl)	0.35	0–0.47	Normal
ALT	24	0–39	Normal
Total Bilirubin	2.48	<1.0	High
Direct Bilirubin	0.69	<0.3	Normal

**FIGURE 1:**
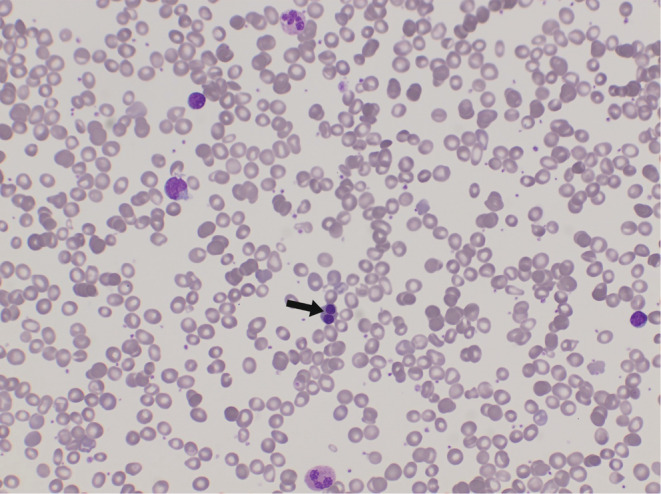
Peripheral blood smear, poikilocytosis, polychromasia, binucleated erythroid. It is not common to see binucleated erythroid in peripheral blood film but luckily, we found it in our patient and guided us to do bone marrow aspirate. (Leishman stain 400X).

Peripheral blood film showed normochromic macrocytic red blood cells (RBCs), marked polychromasia and binucleated erythroid, mild poikilocytosis (ovalocyte, teardrop, microspherocyte), few atypical lymphocytes with normal platelets (Figure 1 **)**.

**FIGURE 2:**
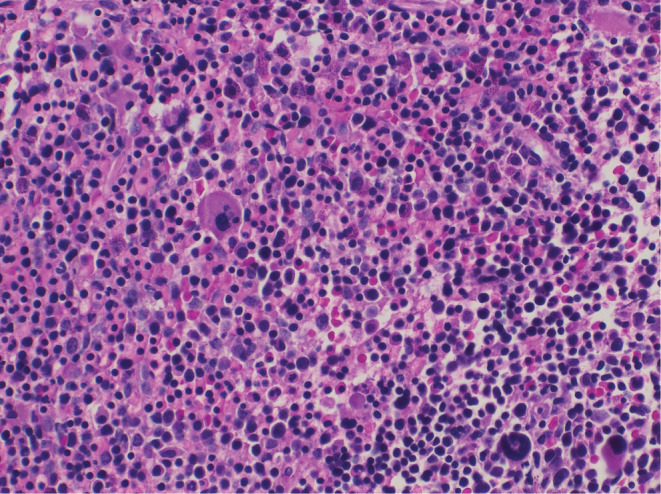
Bone marrow biopsy, from our patient showing erythroid hypercellularity. The biopsy is not significant to confirm the diagnosis of CDA, but we use it to exclude other differential diagnoses of malignancy, hemolytic anemia, or bone marrow failure (H&E stain 400X).

**FIGURE 3:**
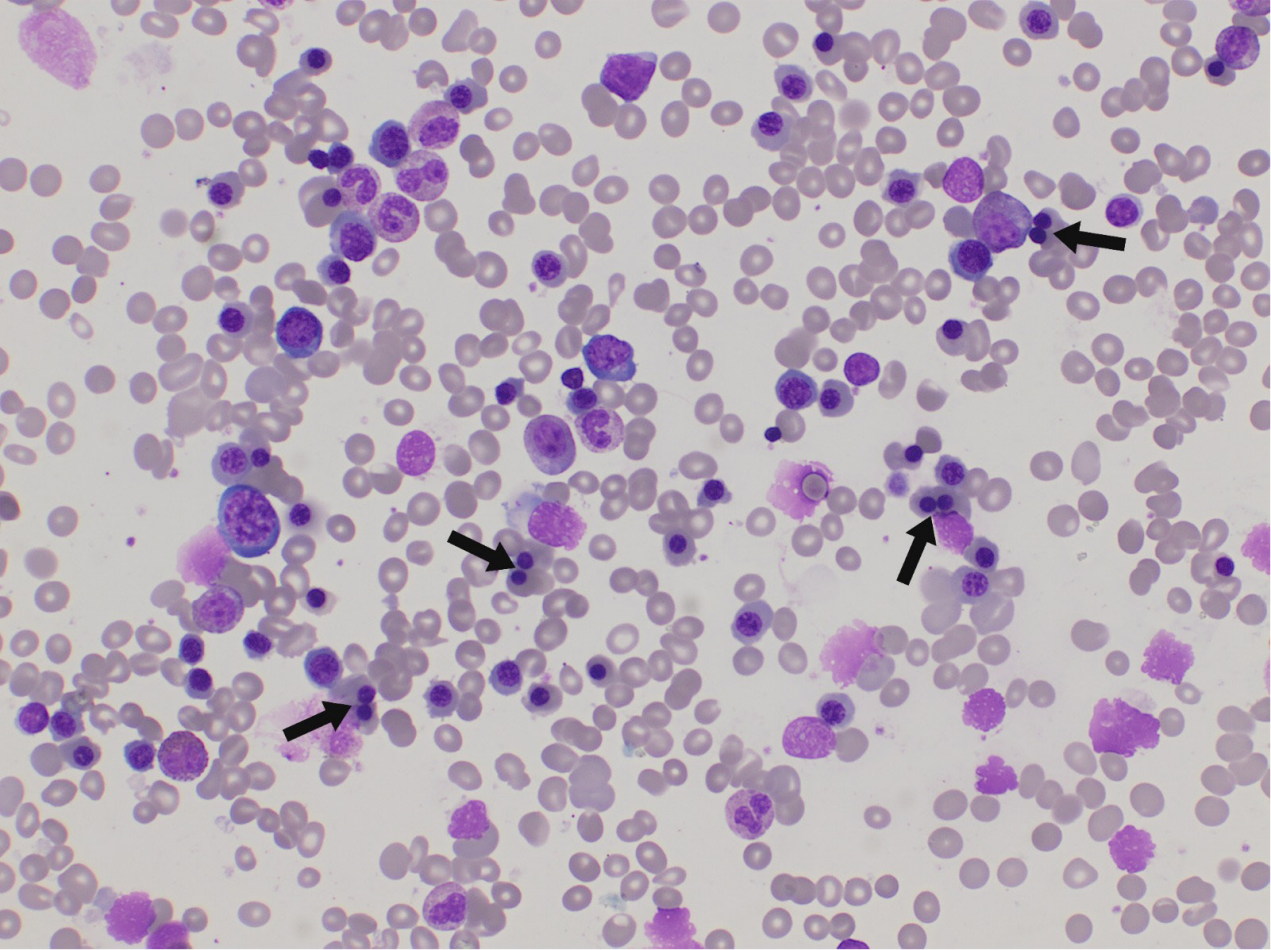
Bone marrow aspirate from our patient, showing erythroid hypercellularity and binucleated forms (black arrows) which are considered the cornerstone of our diagnosis of CDA II (Leishman stain 400X).

Bone marrow smears showed distinct hypercellularity due to erythroid hyperplasia **(**Figure 2 **)**. The myeloid/erythroid ratio was 6:1, and the erythroid precursors were quantitatively markedly increased; also, erythroid maturation was megaloblastic and dyserythropoietic with many binucleated forms **(**Figure 3 **)**. Blasts were not increased, and there was no evidence of fibrosis.

These findings of bone marrow aspirate and laboratory results fit the diagnosis of congenital dyserythropoietic anemia type II.

The management continued with monitoring of the HB/PCV ratio, with Ferritin and blood transfusion when needed. Chelation was not needed because the ferritin was not that high. The genetic study was not done the family refused the test despite a thorough discussion with them about the importance of the test.

## 3 DISCUSSION

An uncommon hematological condition known as congenital dyserythropoietic anemia (CDA) has primarily been documented in Central and Western Europe and North Africa.^1^, ^3^ Congenital dyserythropoietic anemia type one has been previously reported in Jordan in two siblings caused by a homozygous mutation of the C150RF41 gene. They both showed dramatic responses to interferon‐alpha treatment which was maintained for more than 2 years with no toxicity.^4^

Different from other rare anemias, the hallmark of the CDAs is ineffective erythropoiesis and morphological abnormalities of erythroblasts. Other hematopoietic lineages are intact. Also, a hemolytic component is noted. The definition of dyserythropoiesis is the presence of erythroblast abnormalities indicative of aberrant proliferation or differentiation.^5^ The hallmark of the disease is ineffective erythropoiesis as the main feature, and there are distinct morphological abnormalities of the bone marrow's erythroblasts. It should be considered in any patient with chronic anemia. It is classified into three types (CDA I, CDA II, and CDA III), with the most common being type II and CDA III and its nonfamilial type being the rarest. CDA I and CDA II are autosomal recessive in nature, whereas the pattern of inheritance of CDA III and CDA IV is autosomal dominant.^6^ To reach the precise diagnosis of congenital anemias is often delayed. Congenital anemia, jaundice, or hereditary evidence are necessary for diagnosing CDAs. Also, evidence of ineffective erythropoiesis should be present. In addition, the erythroblasts' characteristic morphology is thought to be the key to making the diagnosis, and it is important to rule out congenital anemias, including thalassemia syndromes, hemoglobinopathies, and hereditary sideroblastic anemias. Ineffective erythropoiesis should be suspected if there is inadequate reticulocytosis to the degree of anemia despite erythroid hyperplasia; indirect hyperbilirubinemia and low haptoglobin indicate ongoing intramedullary and extramedullary hemolysis.^6^

Moreover, if appropriate preparation techniques are applicable in bone marrow aspirate, hypercellularity, and distinct erythropoietic hyperplasia are always seen in histobiopsies. By morphological study using light microscopy, CDA I can be diagnosed with great specificity. The most specific finding is the abnormality of chromatin structure with fine chromatin bridges. However, the most specific finding in CDA II is the existence of binucleated cells with two equal‐sized nuclei in each cell. Pseudo‐Gaucher cells that contain birefringent needles can be seen in types I and II.^2^ A mutation of the CDAN1‐gene and/ or typical aberrations seen by electron microscopy are required to confirm the diagnosis of CDA I. The CDAN1‐gene is mapped to chromosome 15q15.1–3. The protein encoded by the CDAN1‐gene was named Codanin‐1. Also, a mutation of the SEC23B‐gene, which is mapped to chromosome 20, is required to confirm the diagnosis of CDA II.^2^, ^3^

Furthermore, iron loading and cholelithiasis are found in all forms of CDA.^6^ Peripheral blood smear in most cases of CDA shows anisopoikilocytosis, mature erythroblasts, basophilic stippling, and poikilocytes.^2^

Differential CDA diagnoses include thalassemia syndromes, some hemoglobinopathies, hereditary sideroblastic anemia, congenital myelodysplasia, and congenital anemia such as Blackfan‐Diamond anemia and Fanconi anemia. Also, other forms of CDA should be taken into account.^1^ Our patient has chronic anemia with chronic blood transfusion. Also, she has splenomegaly with features of extramedullary hematopoiesis. Moreover, a blood smear showed normochromic macrocytic RBCs with mild poikilocytosis and anisopoikilocytosis. The binucleated erythroid was found even in the peripheral blood, there was reticulocytosis with a negative direct coombs test. These laboratory results are commonly not observed in hemoglobinopathies or thalassemia syndromes. Hereditary sideroblastic anemia was ruled out because neither ringed sideroblasts nor dysplastic characteristics on the granulocytic/megakaryocytic lineage were discovered. Lab results do not fit any cause of hemolytic anemia because of high MCV we thought of congenital dyserythropoietic anemia and went for Bone marrow aspirate. Bone marrow aspirate showed hypercellularity, with the erythroid precursors quantitatively markedly increased; also, erythroid maturation was megaloblastic and dyserythropoietic with many binucleated forms. Blasts were not increased, and there was no evidence of fibrosis. These findings of bone marrow aspirate and laboratory results fit the diagnosis of congenital dyserythropoietic anemia type II.

In conclusion, any kid with chronic anemia, hepatosplenomegaly, evidence of extramedullary hematopoiesis, and characteristics of erythroid hyperplasia and dyserythropoiesis in bone marrow aspirate studies should be evaluated for congenital dyserythropoietic anemia. The diagnosis can be made with high accuracy using bone marrow aspirate analysis and peripheral blood smear analysis.

## AUTHOR CONTRIBUTIONS

**Hamzeh F. Al Hussien:** Data curation; writing – original draft; writing – review and editing. **Basil N. Al‐Ekeer:** Data curation; writing – original draft; writing – review and editing. **Hashem Abu Serhan:** Data curation; writing – original draft; writing – review and editing. **Issam Haddadin:** Data curation; writing – original draft; writing – review and editing. **Abdulqadir Nashwan:** Data curation; writing – original draft; writing – review and editing.

## FUNDING INFORMATION

This study was not funded.

## CONFLICT OF INTEREST STATEMENT

The authors declare that they have no competing interests.

## ETHICAL APPROVAL

The article describes a case report. Therefore, no additional permission from our Ethics Committee was required.

## CONSENT

Written informed consent was obtained from the patient's legal guardian to publish this report in accordance with the journal's patient consent policy.

## Data Availability

All data generated or analyzed during this study are included in this published article.
